# Hepatitis E Virus (HEV) Infection in the Context of the One Health Approach: A Systematic Review

**DOI:** 10.3390/pathogens14070704

**Published:** 2025-07-16

**Authors:** Sophie Deli Tene, Abou Abdallah Malick Diouara, Sarbanding Sané, Seynabou Coundoul

**Affiliations:** Groupe de Recherche Biotechnologies Appliquées & Bioprocédés Environnementaux (GRBA-BE), Laboratoire Eau—Énergie—Environnement—Procédés Industriels (LE3PI), École Supérieure Polytechnique (ESP), Université Cheikh Anta Diop (UCAD), Dakar 5085, Senegal; sophiedelitene@esp.sn (S.D.T.); sarbandingsane@esp.sn (S.S.); seynaboucoundoul@esp.sn (S.C.)

**Keywords:** hepatitis E virus, genotypes, environment, transmission routes, human, zoonotic factors

## Abstract

Hepatitis E virus (HEV) is a pathogen that has caused various epidemics and sporadic localized cases. It is considered to be a public health problem worldwide. HEV is a small RNA virus with a significant genetic diversity, a broad host range, and a heterogeneous geographical distribution. HEV is mainly transmitted via the faecal–oral route. However, some animals are considered to be natural or potential reservoirs of HEV, thus elucidating the zoonotic route of transmission via the environment through contact with these animals or consumption of their by-products. Other routes of human-to-human transmission are not negligible. The various human–animal–environment entities, taken under one health approach, show the circulation and involvement of the different species (mainly *Paslahepevirus balayani* and *Rocahepevirus ratti*) and genotypes in the spreading of HEV infection. Regarding *P. balayani*, eight genotypes have been described, of which five genotypes (HEV-1 to 4 and HEV-7) are known to infect humans, while six have been reported to infect animals (HEV-3 to HEV-8). Furthermore, the C1 genotype of the rat HEV strain (HEV-C1) is known to be more frequently involved in human infections than the HEV-C2 genotype, which is known to infect mainly ferrets and minks. Contamination can occur during run-off, flooding, and poor sanitation, resulting in all of these genotypes being disseminated in the environment, contaminating both humans and animals. This systematic review followed the PRISMA guidelines and was registered in PROSPERO 2025 CRD420251071192. This research highlights the importance of investigating the transmission routes and major circulating HEV genotypes in order to adopt a holistic approach for controlling its emergence and preventing future outbreaks. In addition, this article outlines the knowledge of HEV in Africa, underlining the absence of large-scale studies at the environmental, human, and animal levels, which could improve HEV surveillance on the continent.

## 1. Introduction

Hepatitis E virus (HEV) is a public health concern causing large outbreaks and sporadic cases of acute hepatitis around the world [1]. The World Health Organization (WHO) estimated that 20 million people are infected with HEV worldwide each year, resulting in about 3.3 million symptomatic cases of hepatitis E. In 2015, this led to approximately 44,000 deaths, accounting for 3.3% of the mortality due to viral hepatitis [1,2]. HEV infection is the most or second most common cause of acute viral hepatitis among adults in much of Asia, the Middle East, and Africa [3,4]; however, the actual burden of HEV infection remains unknown [5]. HEV is a small single-strand positive-sense RNA virus belonging to the *Hepeviridae* family, which is divided into two subfamilies, namely *Orthohepevirinae* and *Parahepevirinae*, and five genera (*Avihepevirus*, *Chirohepevirus*, *Paslahepevirus*, *Rocahepevirus*, and *Piscihepevirus*) [6]. *Orthohepevirinae* infect mammals and birds, while *Parahepevirinae* infect fish. Ten species have been described to date: *A. magniiecur*, *A. egretti*, *C. eptesici*, *C. rhinolophi*, *C. desmoid*, *P. balayani*, *P. alci*, *R. ratti*, *R. eothenomi*, and *P. heenan* [6,7]. Among the listed species, two have been identified as emerging and therefore of public health concern, namely, *Paslahepevirus balayani* and *Rocahepevirus ratti*. The genetic diversity of *Paslahepevirus balayani* species includes eight genotypes (HEV-1 to HEV-8) with a very heterogeneous worldwide distribution and a broad host range with different prevalence rates depending on the affected animal species, sampling region, or breeding system [8]. It is known that five of the above-mentioned HEV genotypes (HEV-1 to 4 and HEV-7) cause hepatitis in humans [9]. HEV-1 and HEV-2 are found only in humans and are predominant in low-income Asian and African countries. Outbreaks have mainly occurred via the faecal contamination of drinking water due to poor water hygiene and sanitation and contaminated water supply [10,11,12,13]. There is considerable epidemiological evidence of waterborne HEV transmission, especially in Southern/Southeast/Central Asia and Northwest Africa [11,14,15]. HEV-3 and HEV-4 are found in humans and more frequently in animal, including domestic pigs, wild boar, deer, and rabbits [16]. Sporadic cases of autochthonous HEV have been reported in many developed countries by contact with infected animals, mainly after the consumption of raw and undercooked meat products [17]. These include infected pork liver, pork products containing liver, and other pig meat consumed raw or undercooked, which underlines direct evidence of zoonotic HEV transmission [18,19,20]. HEV-5 and HEV-6 were observed among wild boars in Japan; HEV-7 was identified in Dromedary camels in the Middle East; and genotype 8 was identified among Bactrian camels in China [21,22]. Only one human infection with HEV-7 was reported to be due to consuming contaminated camel meat and milk [23]. In addition, recent studies have reported the involvement of the *Rocahepevirus ratti* (rat HEV) species in the zoonotic transmission of HEV, especially in immunocompromised patients and in transplant recipients [24,25,26]. Among rat HEV genotypes (including HEV-C1 and HEV-C2), HEV-C1 has been reported to be responsible for human infections, with the first human case identified in Hong Kong caused by HEV-C1 [27,28]. This genotype also accounts for the majority of human infections reported cases with rat HEV. However, although identified in its carnivorous hosts (ferrets and minks), the zoonotic transmission of HEV-C2 is described as rare and not well studied [29]. Globally, despite reported human cases, the routes of transmission of rat HEV to humans, as well as exposure factors, remain unclear and undetermined. The expanded host range and animal reservoirs indicate these HEV strains’ high variability and zoonotic potential [16,30]. Furthermore, HEV shed by infected animals can contaminate water irrigation sources and lead to the accumulation of HEV in fruits and vegetables [18]. Infectious HEV has also been found in swine manure and wastewater [31]; therefore, applying manure to land and subsequent runoff could contaminate coastal water, leading to shellfish contamination and, subsequently, possible human infection. HEV-3 RNA has been detected in red fruit, strawberries, salad greens, and spices [32], as well as in oysters and mussels [33]. Other transmission routes have also been documented, including blood transfusion and tissue transplantation in immunocompromised patients [34,35,36,37,38,39]. Studies from developed countries have shown asymptomatic viremia (HEV-RNA) in blood donors, suggesting ongoing subclinical infection [40,41]. To mitigate this transmissibility, several developed countries have adopted measures to enhance blood safety based on the epidemiology of HEV [18,42,43]. 

Cases of hepatitis E infection are often asymptomatic, self-limited, and rapidly resolving in immunocompetent individuals. As a result, mortality rates observed in the general population are relatively low, ranging from 0.5 to 4% [44,45,46,47]. However, severe forms of the infection occur in different groups of vulnerable populations, including pregnant women, immunocompromised persons, and people with pre-existing chronic liver disease, leading significant mortality [3,48,49]. High mortality rates of up to 30% have been reported in pregnant women, particularly during the third trimester of pregnancy. Due to their vulnerability, they are more likely to develop complex forms of the disease. In addition, cases of preterm delivery, low birth weight, and stillbirth of the foetus or newborn have been documented [50,51,52]. 

The first documented outbreak of HEV infection in Africa likely occurred in 1950 in Tunisia [53]. In a systematic review of African studies, at least 17 outbreaks were reported in Africa about once every other year since 1979 in 28 countries out of 54, causing a reported 35,300 cases with 650 deaths [45,54]. The most recent epidemics reported in Africa occurred in 2017 (Niger, Nigeria, and Chad) and 2018 (Namibia) [55]. On the other hand, Modiyinji et al. conducted a systematic review of the prevalence of HEV in animals in Africa, highlighting that some animals could be the reservoir of HEV and suggesting the need for molecular epidemiological studies for investigating zoonotic transmission in Africa [56,57].

To date, published reviews in Africa have focused solely on the epidemiology of HEV in humans [54] or animals [56]. To our knowledge and based on our literature search, no previous review in Africa considers human, animal, and environmental factors together in a one health context when studying the epidemiology of HEV. There is a lack of reviews that provide an overview of HEV epidemiology in Africa covering these three aspects. To address these critical gaps, this systematic review aims to explore the epidemiology of HEV from a one health perspective. It will mainly focus on the presence of HEV in drinking water, wastewater, domestic fauna, and the population of blood donors. 

## 2. Methods

**Figure 1:**
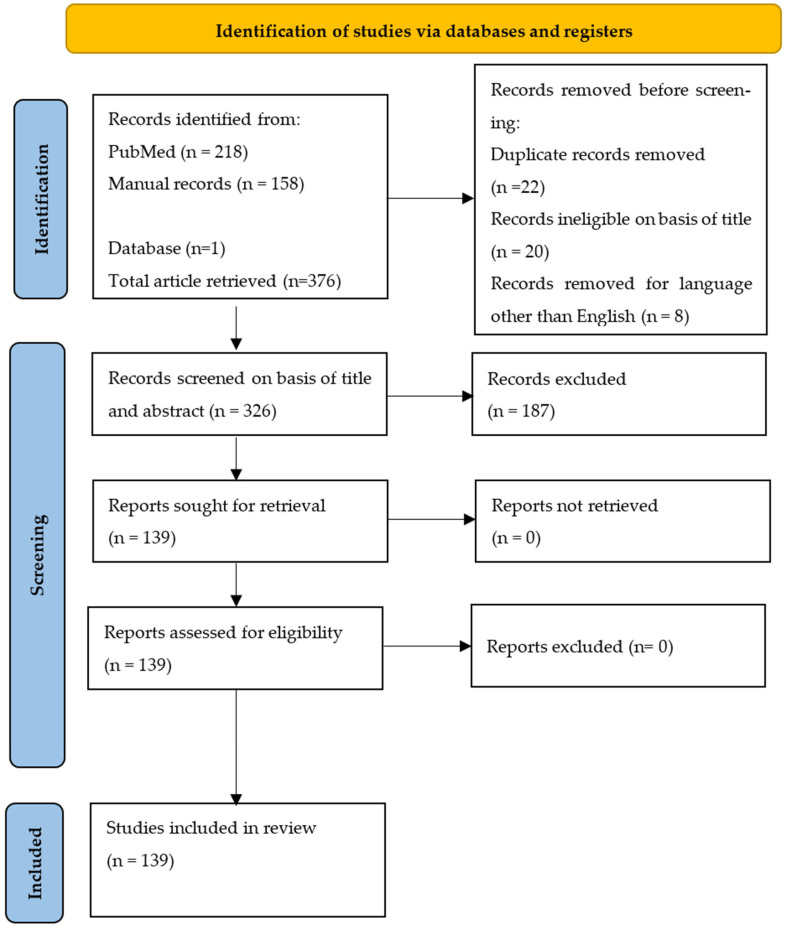
Flowchart summarizing the research process.

A literature search was conducted in PubMed. Systematic reviews, meta-analyses, original articles, and reviews were screened from this database between 2004 and 2024. The following set of keywords was used in combination to retrieve these articles: HEV, Hepatitis E, non-A, non-B, epidemiology, blood donor, food, wastewater, drinking water, and agriculture (Table 1). Only documents published in English with an abstract and full text were used. Articles were excluded based on their title and abstracts or considered irrelevant to this review or related to similar subjects. For manual records, additional keywords such as “one health” and “Africa” were used. For completeness, additional relevant articles have been included, mainly those relating to rat HEV, which is now an emerging cause of hepatitis E infection. The search process is summarized in Figure 1 as a flowchart generated according to the Preferred Reporting Items for Systematic Reviews and Meta-Analysis (PRISMA) guidelines. This systematic review was registered in PROSPERO 2025 CRD420251071192 and is available from https://www.crd.york.ac.uk/PROSPERO/view/CRD420251071192 (accessed on 11 June 2025).

## 3. Taxonomy, Genetic Features and Global Distribution of HEV

HEV is a hepatotropic virus with a genome length of approximately 6.4–7.2 kb. The genomic architecture indicates a single-stranded RNA virus of positive polarity, with a cap of 7-methylguanosine at its 5′ end and polyadenylated at its 3′ end. The genome structure comprises three Open Reading Frames (ORFs) [58]. A fourth open reading frame (ORF4) (embedded within ORF1) present in only genotype 1 strains has also been described and overlaps with ORF1 [59,60,61]. ORF1 at the 5’ end of the genome encodes the non-structural polyprotein, while ORF2 encodes the capsid protein at the 3′ end of the viral genome. ORF3 encodes a small multifunctional, phosphorylated, and palmitoylation protein consisting of 113 or 114 amino acids (aa) [60,62,63] and overlaps with the 5′ end of ORF2. This overlapping region is highly conserved and can be used for the molecular detection of HEV RNA [9,64,65]. Additionally, the 5′ end of the viral genome acts as a binding site for the viral RNA-directed RNA polymerase (RdRP), is also highly conserved, and can therefore be used as a target for molecular detection [66].

In the infected host, HEV exists in two forms: non-enveloped virions (neHEVs) 27–34 nm in diameter when secreted in faeces and bile and quasi-enveloped (eHEV) particles in circulating blood and infected cell culture supernatants [58,67]. The eHEV particles are covered by a lipid envelope, improving bloodstream survival and virion entry, while neHEV is known to be the most infectious form of HEV. This last one is also more stable in bile and faeces environments, enabling transmission via the faecal–oral route [68]. The existence of these two forms allows HEV to evolve in different environments.

The major HEV strain infecting humans is classified in the genus *Paslahepevirus*, belonging to the family *Hepeviridae*, subfamily *Orthohepevirinae* [7], and can be further divided into two species: *Paslahepevirus balayani* (formerly known as *Orthohepevirus* A) and *Paslahepevirus alci* [8,69].

**Figure 2:**
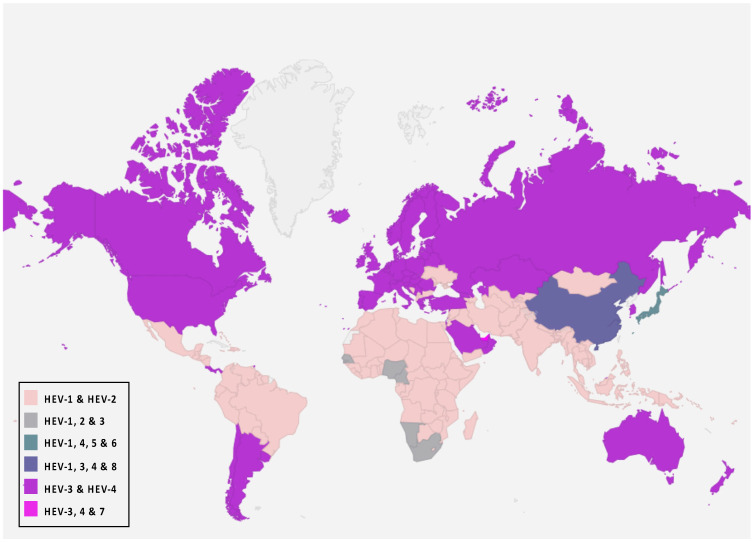
*P. balayani* global distribution according to genotypes.

Eight genotypes have been identified in the *P. balayani* species, five of which are reported to cause human disease [70,71]. HEV-1 and HEV-2 genotypes are widely dis-tributed. These strains are pathogenic only for humans and are endemic in developing countries where they are responsible for significant waterborne outbreaks in endemic regions of Africa, South and Southeast Asia, and Mexico [61,72]. HEV genotypes 3 and 4 (HEV-3 and HEV-4) are zoonotic strains with animal reservoirs, and a broad host range is known (pigs, cattle, sheep, goats, horses, macaques, cats, dogs, rabbits, mongoose, rats, and mice) and they have a geographical spread around the world. These genotypes of zoonotic HEV cause sporadic and cluster cases in both industrialized and developing countries [44,58,72,73]. Furthermore, local outbreaks caused by HEV-3 and 4 have been reported [74,75,76,77,78]. They can be transmitted to humans mainly through consuming contaminated foodstuffs, including raw and undercooked meat products from the animals mentioned above, because they circulate in both domestic and wild fauna. In addition to the zoonotic transmission route, many studies reported that these genotypes could also be transmitted parenterally via blood and blood products [17,19,79,80]. Two unrecognized and different new genotypes were isolated from Japanese wild boar for the first time and identified as genotypes 5 and 6 [81,82]. In addition to these, two additional HEV genotypes have been identified in dromedaries (DcHEV) and camels (BcHEV), respectively, HEV-7 and HEV-8, further expanding the host diversity of HEV [22,72,83]. These camel HEV genomes possessed >20% nucleotide difference from all other HEVs with complete genome sequences available [84]. HEV-7 is likely to be a causative agent for human hepatitis E infection and has been identified in a man who regularly consumed camel meat and milk [23,82,85,86]. It is important to note that HEV-7 and HEV-8 genotypes were identified in the Middle East and China, respectively. However, the knowledge and geographical distribution of these genotypes remain limited compared to the other genotypes. Figure 2 shows the global distribution of *P. balayani* genotypes and their overlaps in specific geographical areas.

**Figure 3:**
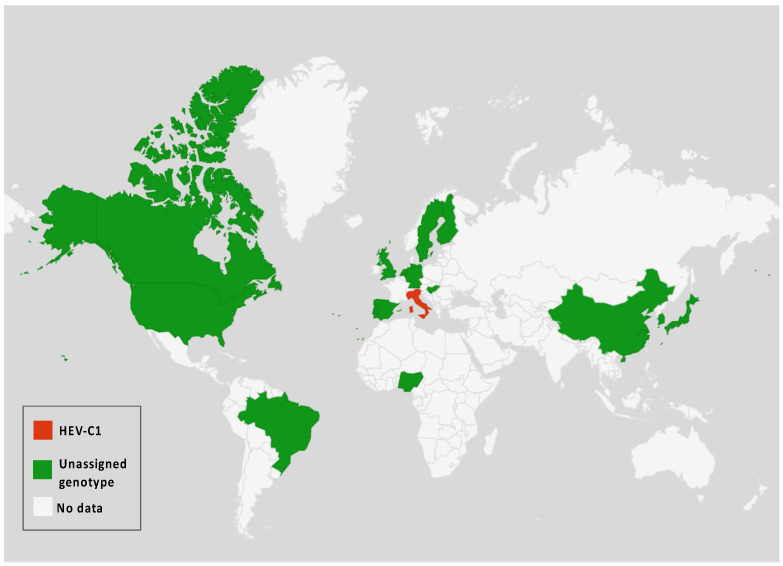
*Rocahepevirus* genus global distribution according to genotypes among humans and animals.

In addition to the common *Paslahepevirus balayani* strains causing hepatitis E infections in humans, an emerging HEV species, *Rocahepevirus ratti* (classified initially as *Orthohepevirus C*), which is genetically very different, has been identified primarily in rats (Rat-HEV) and other rodents (shrews, ferrets, minks, and wild rodents) [87]. This species has only 50–60% genomic identity and a significant ORF2 sequence divergence compared to *P. balayani*. Its intra-species diversity shows two main genotypes known as HEV-C1 and HEV-C2, with rodents and carnivores as their respective hosts. Rat HEV strains primarily infect rats but have also been identified as capable of crossing the species barrier and infecting humans, thus presenting a potential risk of zoonotic transmission. However, although HEV-C1 is most frequently involved in human infections, the C1 genotype predominantly comes from rats, while C2 strains originate from ferrets and minks. In addition, two other putative genotypes have been proposed to date, namely C3 and C4 [25], but have yet to be assigned (Figure 3).

The genomic structure of *P. balayani* is similar to that of other hepeviruses, including Rat-HEV [88], except a single putative ORF identified in some rat HEV strains, the function of which is unclear [89].

## 4. Phylogenetic Analysis

**Figure 4:**
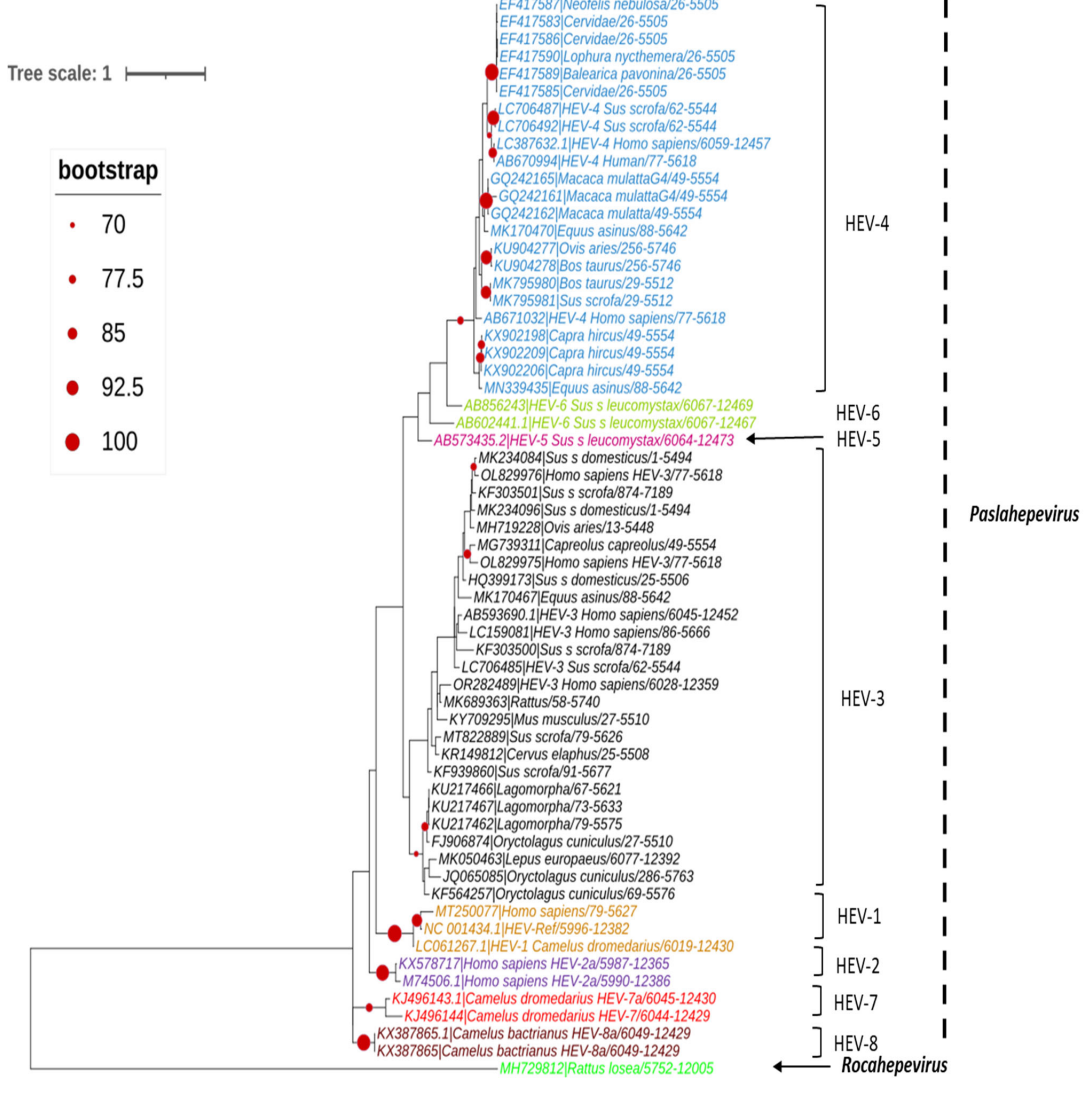
Phylogenetic tree describing the genetic diversity of P. balayani. A maximum likelihood (ML) tree [based on the genomic region 5995–6226 relative to NC_001434 reference sequence] showing the relationships between HEV genotypes based on partial sequences (ORF-2 capsid). The tree was constructed using 62 isolates (with tags: Accessions number_host_genotypes) under the GTR nucleotide substitution model with 4 Gamma categories using PhyML [91] on seaview software v5.0.4 [90]. For branch support, bootstrap values between 70 and 100% are indicated by red dots.

**Figure 5:**
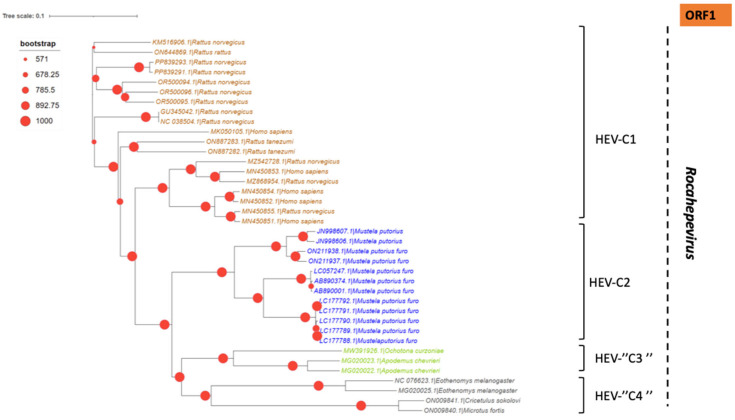
Phylogenetic tree describing the genetic diversity of *R. ratti* based on ORF-1. A maximum likelihood (ML) tree [based on the genomic region 42–4921 relative to NC_038504.1 reference sequence] showing the relationships between HEV genotypes based on partial sequences (ORF-1). The tree was constructed using 37 isolates (with tags: Accessions number_host_genotypes) under the GTR nucleotide substitution model with 4 Gamma categories using PhyML [91] on seaview software v5.0.4 [90]. For branch support, bootstrap values between 571 and 1000 are indicated by red dots.

**Figure 6:**
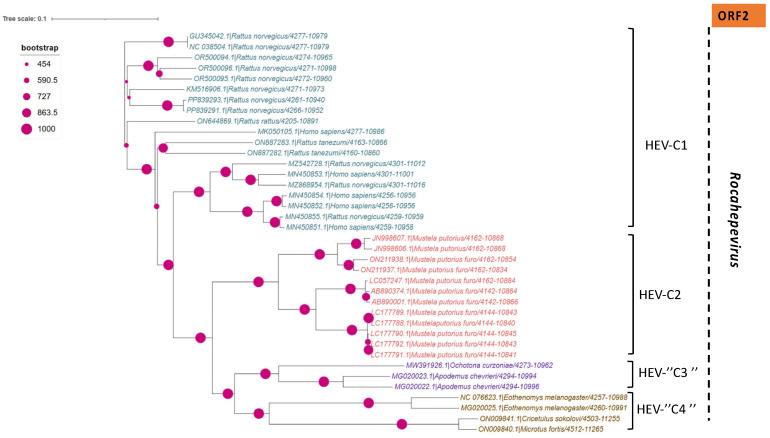
Phylogenetic tree describing the genetic diversity of *R. ratti* based on ORF-2. A maximum likelihood (ML) tree [based on the genomic region 4949–4921 relative to NC_038504.1 reference sequence] showing the relationships between HEV genotypes based on partial sequences (ORF-2). The tree was constructed using 37 isolates (with tags: Accessions number_host_genotypes) under the GTR nucleotide substitution model with 4 Gamma categories using PhyML [91] on seaview software v5.0.4 [90]. For branch support, bootstrap values between 454 and 1000 are indicated by magenta red dots.

**Figure 7:**
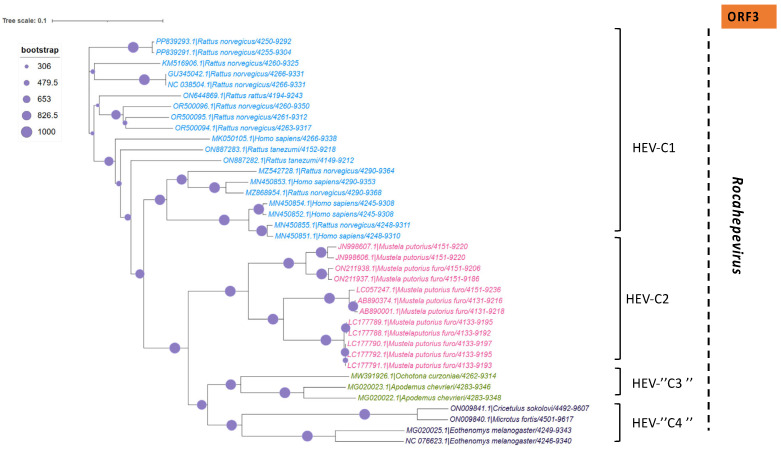
Phylogenetic tree describing the genetic diversity of *R. ratti* based on ORF-3. A maximum likelihood (ML) tree [based on the genomic region 4938–5246 relative to NC_038504.1 reference sequence] showing the relationships between HEV genotypes based on partial sequences (ORF-3). The tree was constructed using 37 isolates (with tags: Accessions number_host_genotypes) under the GTR nucleotide substitution model with 4 Gamma categories using PhyML [91] on seaview software v5.0.4 [90]. For branch support, bootstrap values between 454 and 1000 are indicated by purple dots.

A set of HEV full-length, near full-length genomes and partial sequences of HEV (ORF-2 for *Paslahepevirus* genus) and (ORF-1, ORF-2 and ORF-3 for *Rocahepevirus* genus) were downloaded from GenBank for phylogenetic reconstruction purposes. For each set, all sequences considered are reduced to the same size and cover the genomic region from the nucleotide position [5995 to 6226 (ORF-2) for the *Paslahepevirus* genus, then 42-4921 (ORF-1), 4949-6883 (ORF-2), and 4938-5246 (ORF-3) for the *Rocahepevirus* genus] relative to the NC_001434 and NC_038504.1 reference sequence respectively for the *Paslahepevirus* and *Rocahepevirus*. Then they are subject to multiple sequence alignment with MUSCLE and gap positions are removed by using the Gblocks program on SEAVIEW software v5.0.4 [90]. Phylogenetic trees were inferred by the maximum likelihood method with PhyML v3.1 [91]. The trees were generated under the best-fit nucleotide substitution model GTR + G + I determined by MODELTEST in MEGA X [92]. The Subtree-Pruning-Regrafting heuristic search was applied for optimal tree topology. Branch supports were determined with 100 bootstrap replicates. The phylogenetic trees were read and edited with Figtree [93] (Figure 4, Figure 5, Figure 6 and Figure 7).

## 5. Transmission Routes of HEV

**Figure 8:**
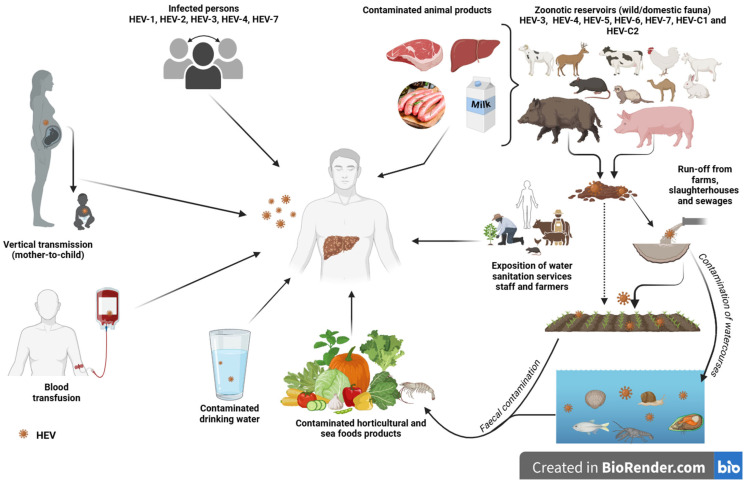
Different transmission routes of hepatitis E virus (created with BioRender.com, accessed on 27 June 2024).

Multiple transmission routes are enabling the spread of hepatitis E virus. However, the two main routes of transmission are faecal–oral and zoonotic in developing and industrialized countries, respectively. In addition to these, with all the contaminations that occur directly or indirectly, mother-to-child transmission, human-to-human transmission, and transmission through blood transfusions are often reported and are therefore not negligible. In Figure 8, we summarize the different transmission routes known to date for the circulation of HEV in the environment, in humans and in animals recognized as natural reservoirs of the virus.

### 5.1. Environment

#### 5.1.1. Drinking Water

Waterborne diseases threaten human health, particularly in low-income countries [94]. According to the WHO, in 2022, at least 1.7 billion people were using drinking water sources contaminated with faeces. The bacterial and viral contamination of drinking water due to faecal contamination poses the most significant risk to drinking water safety [95].

HEV infection is often a waterborne disease associated with epidemics and sporadic cases worldwide, frequently due to HEV genotypes 1 and 2. Hepatitis E virus transmission to humans through water has been largely demonstrated for these genotypes, primarily in developing countries [96]. The leading causes of outbreaks were limited access to or the contamination of drinking water, inadequate sanitation, hygiene, and health services, and the poor disposal and treatment of sewage. In addition to these most frequently reported phenomena, overpopulation, the lack of drinking water, and periodic flooding have been identified as contributors to the hepatitis E outbreak in Southern Sudan in 2023 [97]. A recent systematic review based on a meta-analysis reported a worldwide prevalence of HEV in drinking water of 4.7% [57].

Water transmission is also suspected for the zoonotic genotypes since HEV-3 and HEV-4 have been detected in different environmental waters [96,98,99,100]. An investigation of a tap water-mediated HEV outbreak in China suggests that sporadic cases of waterborne outbreaks of HEV-4 may occur in industrialized countries [101,102].

#### 5.1.2. Wastewater and Surface Water

HEV has been detected in wastewater worldwide, particularly in developing countries, where sanitation and water supply services remain precarious or inaccessible for some populations. HEV is an enteric virus that can be excreted as a non-enveloped particle in the faeces of humans (wastewater) and animals (runoff from slaughterhouses, pig farms, or free-ranging animals) [103]. The faeces shed the virus in high amounts, with a documented amount of up to 10^11^ viral particles per gram of stool [104,105] and a median peak of approximately 10^10^ genome copies/g [106,107].

Most of the time, the faeces of HEV-infected animals and humans can reach sewer systems through environmental phenomena such as flooding in rainy seasons, then contaminating municipal wastewater treatment plants (WWTPs), therefore potentially increasing the spread of the virus in the environment [100,102]. On the other hand, livestock and/or slaughter waste (blood, water, faeces, etc.) from these animals, which constitute a zoonotic reservoir for HEV, end up in wastewater in some way. As a result, the inadequate treatment of this water could lead to the severe contamination of the environment and, hence, the emergence of the disease in humans exposed to this wastewater. Usually, these people are the water sanitation and supply services staff. They are at a significant risk of exposure to HEV [108].

Coastal waters can become contaminated by human sewage and manure runoff, and therefore, HEV can accumulate in certain organisms produced close to land [102,109,110,111]. 

Until 2019, water’s role in transmitting HEV-3 had only been suspected [98]. Given its zoonotic nature, detecting the viral genome in wastewater concerns public health authorities [112].

The global HEV prevalence in water matrices was estimated to be about 15.14% in untreated wastewater, 3.81% in treated wastewater, and 7.46% in surface waters [57]. According to this systematic review, the most predominant genotypes found were HEV-3, followed by HEV-1 and HEV-4. In addition, authors report that HEV in water environments found in Europe (12.2% [7.6–17.7]), Asia (9.9% [0.7–26.2]) and America (6.5% [0.9–15.4]) was all higher than what was found in Africa (0% [0–3.6%]) [57]. This relatively low prevalence of HEV in wastewater in Africa is likely an underestimation, as confirmed by the limited number of studies (n = 03) on HEV testing in wastewater from 1995 to 2020. A recent study in Cameroon revealed an HEV prevalence of 1.9%, and phylogenetic analysis showed that it belonged to genotype 3, subtype 3a, forming a cluster with swine strains [113]. Most recently, in South Africa, HEV was detected in 21.8% (117/536) of the water samples, with detection rates of 22% (72/328) in wastewater influent, 22% (42/188) in river water, and 10% (2/20) in ablution runoff/standpipe site samples. Sequence genotypes belonged to HEV-3 (subtypes 3c and 3f) and HEV-4 (subtype 4b) with the following rates: 98% and 2%, respectively [114].

Furthermore, while the transmission routes of rat HEV are poorly understood, the virus was detected in French urban wastewater samples (11/11), indicating a significant prevalence of *Rocahepevirus ratti* in the human environment, particularly in urban wastewater [115]. Based on this reported detection, the contamination of wastewater by human faeces, particularly through the existence of asymptomatic cases, must be raised. However, the most plausible hypothesis seems to be the implication of the natural host, mainly rodents, in the sewage system through their dejections, which may reach the urban WWTP. In any case, the detection of *Rocahepevirus* in the environment through wastewater is likely to have a significant impact on its potential as a new source of human contamination as well as its subsequent environmental spreading [115,116]. 

### 5.2. HEV in Foodstuffs

Viruses in food come from the human or animal gastrointestinal tract via excreta and can contaminate water, food, and the environment [13]. Food contamination can occur through direct contact with infected persons or indirectly through contaminated environments, including using contaminated water or faecal matter as fertilizers during food production [107]. 

Although viruses do not multiply in food, HEV is stable under specific physicochemical conditions, persists for months, and remains infectious [117,118]. According to a recent study, the HEV-3 genotype can survive for a long time at 4 °C, and its infectivity in food was reported to remain at −20 °C, even after 12 weeks of storage. The complete inactivation of HEV-3 has been performed by heating at 56 °C for 1 h or at a higher temperature for a shorter time [119]. On the other hand, studies have also demonstrated that HEV remains stable at 56 °C, and temperatures above 71 °C have been shown to completely inactivate HEV, depending on the genotype and food matrices. Indeed, heating a faecal suspension containing HEV-1 and/or HEV-2 at 71 °C results in complete HEV inactivation. 

Furthermore, frying or boiling pork contaminated with HEV-3 and HEV-4 at 191 °C for 5 min ensures complete HEV inactivation in 0.5 to 1.0 cm^2^ pieces of pork liver. The condition mentioned above (191 °C, 5 min) enables core cooking at an internal temperature of around 71 °C, without burning the tissue. These data are essential, as their application on both industrial and household scales will help guarantee food safety, particularly for meat products, to limit the foodborne transmission of HEV as much as possible [120,121]. Foodborne HEV outbreaks are often associated with zoonotic HEV-3 and HEV-4 strains and are transmitted via the faecal–oral route [30,122].

#### 5.2.1. Seafood Products

As a result of waterborne transmission, contaminated, untreated water and farm and slaughterhouse runoff are potential vehicles of pathogens. Via flooding and other natural phenomena, these waters join watercourses and contaminate shellfish production areas and other aquatic areas [57], mainly when these production areas are located near urban or livestock areas [123].

The consumption of fish and seafood products, particularly edible lamellibranch molluscs (ELMs), is a risk to human health because of their capacity to filter, accumulate, and concentrate pathogens present in the water within their tissues. In certain countries, bivalves and shellfish are tested regularly for faecal contamination using *E. coli* as an indicator, which is poor for reporting the presence of viral faecal contamination [124]. 

Shellfish consumption has been identified as a risk factor for hepatitis E in several case reports [125,126] and caused an outbreak of hepatitis E aboard a cruise ship [75]. Bivalves are widely recognized as one of the foods most at risk for HEV contamination, especially when consumed raw or undercooked [117,127]. HEV can also persist in shellfish and marine environments for weeks to months [128,129].

Around the world, several studies have documented high HEV positivity rates in shellfish bivalves, mainly in Asian countries, including China, Japan, and Vietnam [130,131,132] and other countries such as the United Kingdom and Spain [33,111,123]. In South Italia, HEV was detected in sampled shellfish in 1 out of 108, accounting for a positivity rate of 0.9% [105]. A positivity rate of 2.9% (9/310) was also reported in shellfish purchased from local markets in Scotland [133], thus highlighting the possible occurrence of HEV infection in shellfish consumers. In addition, a long viral persistence has been demonstrated in these marine animals, making shellfish significant vectors of enteric diseases, particularly HEV [123,134,135].

#### 5.2.2. Fruit and Vegetables

Fresh vegetables and ready-to-eat (RTE) salads have become increasingly recognized as potential vehicles for foodborne diseases [136,137]. The contamination of fruit and vegetables by enteric viruses can occur during cultivation, before the pre-harvest and post-harvest stages, i.e., from farm to fork. Certain cultivation practices, such as fertilization with faecal matter, methods of watering horticultural produce, and water contaminated by faecal matter used as an input during irrigation, are all major contributors to produce contamination. This transmission route may occur due to insufficient initial washing, inadequate cooking practices, inadequate hygiene by food handlers, and specific cultivation and harvesting methods. In brief, major contamination routes of fresh produce are irrigation water and improper food handler hygiene. 

If irrigated with contaminated water, vegetables and berries can retain microbial agents on their surfaces, including enteric viruses [105,138,139,140,141,142]. Therefore, they play an important role in determining environmental and food contamination [105]. 

HEV was found once in a pack of frozen raspberries taken from a cold room at the point of sale with a reported prevalence of 2.6% [143]. In Europe, Kokkinos et al. found the presence of HEV in 5% of irrigation water samples tested from a leafy green vegetable production chain and in 4 out of 125 fresh lettuce samples, accounting for a positivity rate of 3.2% [32], demonstrating that irrigation water is a significant risk factor for HEV transmission in fresh fruit and vegetables; Purpari et al. reported HEV in vegetable samples with a positivity rate of 1.4% from Sicily, Italia [105]. Other authors have also reported for the first time the detection of swine HEV (HEV-3) in strawberries grown in a field where the irrigation water came from a contaminated river located in agricultural and residential areas. The detection of HEV-RNA in fruits and vegetables underlines the risk of exposure related to the consumption of these products, which are frequently eaten raw. Although less frequent than waterborne epidemics, isolated or clustered cases linked to the ingestion of contaminated fruit and vegetables are possible but do not lead to major outbreaks and are therefore not significant.

#### 5.2.3. Animal Products, Including Pork and Liver Products

In industrialized countries, most sporadic cases of hepatitis E infection occur after the ingestion of contaminated meat. Domestic pigs and wild boars are recognized as the primary animal reservoirs of HEV worldwide. Epidemics of HEV have been reported in Europe, particularly in France and Spain, where cases of infection were directly and respectively associated with the consumption of spit-roasted piglets and wild boar [144,145]. Along with the species mentioned above, others are known to be reservoirs carrying hepatitis E virus, including domestic and wild ruminants [146,147]. Anti-HEV antibodies have been detected in other animals, including deer, moose, rats, dogs, cats, camels, mongooses, cows, sheep, goats, avian species, rabbits, bats, and horses. Transmission from animals to humans is also documented through direct and indirect evidence in many countries [14,146,147,148,149,150,151]. 

HEV infection in pigs typically occurs early in life, with peak virus shedding occurring around 3 months of age. In pigs, HEV infection is asymptomatic, short-lived, and rapidly resolves. Indeed, HEV prevalence in pigs varies with age [152]. Among others, a study conducted in pigs in the United Kingdom revealed that the mean prevalence of HEV in pigs aged 3–5 weeks, 10–12 weeks, and 22–24 weeks and in dry adult sows was 26.0%, 44.0%, 8.9%, and 6.0%, respectively [31]. Beyond 12 weeks of life, viremia gradually decreases, showing the importance of considering the slaughter age of pigs.

Several concordant studies across the world have reported the presence of HEV in pigs, suggesting the existence of a zoonotic reservoir and evidence of foodborne transmission [70,126,153,154,155]. In Africa, a review conducted in 2020 by Bagulo et al. has reported high HEV seroprevalence in pigs, ranging from 47 to 80% [12,156,157,158,159,160]. More recently, in Sierra Leona, a low prevalence of HEV in pig sera of 4% (44 out of 1086) was documented, and the circulating strain belonged to genotype 3 [161]. However, a high prevalence of 47.7% of HEV antibodies and 36.1% of HEV-RNA in pigs was obtained, respectively, in Cameroon in 2020 and in the Central African Republic in 2024, still underlining the major circulation of HEV-3 strains in the swine population [162,163].

Pork and pork liver products are the most obvious sources of foodborne HEV and have been extensively studied in Europe. HEV has been detected in pig liver products sold in France, particularly in dried salted pig liver and figatelli, with a reported prevalence of 3% and 30%, respectively [164]. In America, authors have also reported the prevalence of HEV in pork liver products purchased from Ottawa grocery stores, with 10.5% and 47%, respectively, in pork livers and pork pâtés [165]. Few studies have reported HEV as a foodborne pathogen in Africa, mainly in pork products. Therefore, this virus was detected in 2/144 pork liver samples from South Africa in 2014 [166]. Recently, in Senegal, the first study reported the detection of HEV-RNA in pork meat and liver sold in Saint-Louis with a global prevalence of 5.4% with the following positivity rates: 3.1% and 22.2%, respectively, for meat and pork liver [155].

The high prevalence of HEV in pigs’ livers is interpreted as evidence based on the fact that the liver is the organ in which hepatitis E virus carries out its replication cycle [155,167]. The risk of contamination is lower for meat consumption. Still, it remains an important route through which sporadic cases of hepatitis E infection can occur in both developed and developing countries.

Regarding rat HEV, besides the suggested evidence of direct contact with rats or rat droppings, its foodborne transmission has not yet been investigated or reported [115]. Further studies are needed to address this question for a proper and detailed comprehension of their spreading and zoonotic transmission pathway. 

### 5.3. HEV in Blood Products (Or Among Blood Donors)

Besides cases of the waterborne and zoonotic transmission of HEV, several authors have reported cases of transfusion-transmitted HEV, with a constant increase [39]. Cases of acute infection are self-limited in immunocompetent individuals. HEV infection often causes asymptomatic viremia in these individuals and can, therefore, be transmitted to humans via blood transfusion through the symbolic elements of the donor’s blood. Thus, the risk of infection by transfusion is associated with viremia in asymptomatic donors. In the case of HEV, the viremic period is estimated to be up to 88 days [168,169]. However, the probability of developing hepatitis E infection is strongly correlated with the donor’s viral load. In their systematic review, Dreier et al. reported that all components with a viral load > 5.00 × 10^4^ UI cause hepatitis E infection, and this is independent of the recipient’s immune status [170,171]. 

Transmission through blood donation was also documented, with annual observations increasing, representing more than 2.5% of all transmissions [172]. However, it is essential to emphasize that blood transfusions are administered to patients only. The first transfusion-transmitted HEV infection (TT-HEV) was reported in Hokkaido, Japan, in 2004 [173]. Since this first report, more cases have been reported in Japan [174,175], France [36,176,177,178], the UK [34,41], Germany [179], and Spain [180,181]. In all cases, the HEV genomic sequences of the donor and the recipient patient were perfectly similar, confirming the possibility of the transmission of HEV by transfusion.

In several countries, cases of transmission by transfusion are often not reported, not because they do not exist but because they are not systematically diagnosed during blood donation. This could cause severe and chronic infections in immunocompromised recipients or those with certain vulnerabilities. Indeed, it has been reported that an estimated 60% of cases of HEV-infected immunocompromised patients develop chronic hepatitis E [182].

For this reason, some European countries and Japan have introduced screening for HEV RNA in blood donors using a universal screening method for all donors (used in Ireland, the UK and the Netherlands) and a selective screening method (used in France, Austria and Luxembourg), which consists of selecting from blood banks only those products intended for individuals considered to be at a high risk of developing complications linked to transfusion-transmitted HEV infection [171,180]. Analytic approaches such as the mini-pool nucleic acid test (MP-NAT) and individual donation nucleic acid test (ID -NAT) are two possible protocols used for screening HEV in blood supplies; thus, practices vary widely across laboratories worldwide. The latter is more sensitive but more expensive than the MP-NAT, making it challenging to use the ID-NAT in large-scale HEV screening programs [169,180]. 

More generally, these screening methods are often not systematically applied in emerging and developing countries due to a significant lack of resources. This increases the risk of the recurrence of chronic HEV infections, mainly in immunocompromised individuals, including HIV-infected persons, patients with rheumatologic disorders, and patients with haematologic malignancy [61]. 

In most African countries, blood donors are routinely tested only for HIV and hepatitis B and C, not for other emerging or unknown pathogens that could potentially threaten blood safety [157,183]. Due to dietary habits and other factors, all blood donors are exposed to HEV. The regular consumption of pork meat and shellfish was also reported in the viremic donors in China [39,184].

Worldwide, HEV immunomarkers have been investigated among blood donors. Anti-HEV IgG was found in this population group, with seroprevalences ranging from 2% (Fiji) to 43.5% (Poland). Between 1996 and 2018, anti-HEV IgM seroprevalences ranged from 0.2% in India to 49% in Italy [180]. In the same way, HEV RNA was detected among blood donors between 2004 and 2018, and reported positivity rates ranged from 0 to 1:27 (3.7%) in 2004 in Indian blood samples obtained from asymptomatic donors. After sequencing all HEV RNA-positive samples, it was concluded that the HEV-1 genotype was the causal agent of HEV transfusion-associated infections in that study [180,185].

A review of HEV in sub-Saharan Africa reported an overall seroprevalence among blood donors ranging from 0 to 49% and seroprevalences of 4.6 to 47.8% and 3.1 to 5.9% for IgG and IgM, respectively, showing the risk of contamination associated with blood donations [157,160,183,186,187,188,189,190,191]. 

Given that the serological prevalence of HEV is increasing worldwide and that severe cases can occur in recipients of donated blood, screening blood donations for HEV can help to mitigate the risk of transmission and protect vulnerable groups who are more likely to develop chronic forms of hepatitis E disease.

## 6. Environmental-Human–Animal Health Interconnections: One Health Perspective

As described in the previous sections, HEV is capable of surviving and/or persisting in different environments and different forms. In addition, its potential to cross the species barrier means that it has a wide host range, including humans and various animal species, thus revealing its zoonotic potential. 

Under the prism of the “one health” concept, it is important to summarize the transmission pathways in a coordinated and comprehensive approach to take a global view of the various entities involved in the interactions. 

Human or animal faeces containing HEV contaminate the environment. During flood episodes and to avoid possible overflows, water is often discharged into drainage systems, carrying any viral pathogens that may be present [124]. As a result, contaminated wastewater from sewage joins watercourses and aquatic environments and then contributes to the spread of microbial contaminants into the environment. It has been evidenced that sewage can contaminate drinking water sources, thus compromising consumer health and highlighting the risk of infection [192].

On the other hand, the detection of zoonotic HEV in surface and drinking water indicates the potential spreading of HEV-3 through water transmission [193,194,195]. Moreover, surface waters can be contaminated near pig production facilities, mainly due to run-off waters and percolation events or following the agronomic use of pig slurry [57,196,197]. Farm or abattoir run-off, combined sewer overflows, and inadequately treated sewage could be polluting watercourses with HEV [124], therefore offering evidence of thew spreading of the zoonotic genotypes through the environment. Usually, these genotypes are found in animal products. Thus, their detection in water suggests contamination by animal faeces or run-off from domestic farms. Similarly, the species present in the corresponding environments are likely to be contaminated. The persistence of hepatic and enteric viruses in the environment is evidence of their presence in irrigation water, compromising the safety of horticultural products and the consumer’s health [198].

Occupationally exposed humans, including those working in slaughterhouses, forestry workers, hunters, farmers, or veterinarians, have higher HEV and are exposed in various ways to hepatitis E virus, especially as they are often in contact with animals, sewage, and faeces, among other aspects. This provides evidence for zoonotic transmission from animals to humans through direct/indirect contact with animal reservoirs, which also constitutes a high risk of exposure to HEV [148,199]. To control and manage this risk of exposure, it is crucial to pay close attention to hygiene measures and the correct and systematic use of personal protective equipment. In addition, implementing suitable measures for the proper management of animal waste (including wastewater) from farms should be considered.

From the one health perspective, surveillance is necessary to prevent HEV infection and control its emergence or re-emergence in some regions, such as sub-Saharan countries. This approach aims to be collaborative, holistic, and multidisciplinary, guaranteeing and improving human, animal, and environmental health by limiting the global spread of HEV.

## 7. Conclusions

This review provides an overview of current knowledge about HEV and traces the various routes of transmission known to date. The prevalence reported in different matrices (food and environmental samples) and among blood donors highlights the different risk factors for exposure to hepatitis E virus, as well as its circulation within different entities. A substantial part of the population is exposed to HEV through dietary habits, particularly undercooked or uncooked meat products, including shellfish, pork, and pork liver products. Rats, small ruminants and other livestock are also not negligible reservoirs of HEV. In addition, sanitation workers, slaughterhouse workers, and farmers are also at risk of contracting HEV infection. Beyond the fact that HEV is an emerging pathogen with global distribution and should receive more attention from public health authorities, it remains a virus that has been very little studied in Africa. Consequently, large-scale studies should be addressed to further document the genotypes circulating in the continent and understand their interactions with hosts, including those potentially released into the environment, in order to prevent and monitor their spread.

## Figures and Tables

**Table 1:** Search strategy.

Strategies	Set of Keywords
1	“HEV” OR “Hepatitis E” OR “Non-A Non-B”
2	“epidemiology” AND “Hepatitis E virus”
3	(“blood donor” OR “food” OR “wastewater” OR “drinking water” OR “agriculture”)
4	“genotype” AND “Hepatitis E virus”
5 (1 AND 2)	“HEV” OR “Hepatitis E” OR “Non-A Non-B” AND “epidemiology”
6 (5 AND 3 AND 4)	“HEV” OR “Hepatitis E” OR “Non-A Non-B” AND “epidemiology” AND (“blood donor” OR “food” OR “wastewater” OR “drinking water” OR “agriculture”) AND “genotype”

## Data Availability

The original contributions presented in this study are included in the article. Further inquiries can be directed to the corresponding author(s).
